# Apparent diffusion coefficient cannot discriminate metastatic and non-metastatic lymph nodes in rectal cancer: a meta-analysis

**DOI:** 10.1007/s00384-021-03986-8

**Published:** 2021-06-29

**Authors:** Alexey Surov, Hans-Jonas Meyer, Maciej Pech, Maciej Powerski, Jasan Omari, Andreas Wienke

**Affiliations:** 1 Department of Radiology and Nuclear Medicine, Otto-von-Guericke University Magdeburg, Magdeburg, Germany; 2 Department of Diagnostic and Interventional Radiology, University of Leipzig, Leipzig, Germany; 3 Institute of Medical Epidemiology, Martin-Luther-University Halle-Wittenberg, Biostatistics, and Informatics, Halle (Saale), Germany

**Keywords:** Rectal cancer, DWI, ADC, Lymph node

## Abstract

**Background:**

Our aim was to provide data regarding use of diffusion-weighted imaging (DWI) for distinguishing metastatic and non-metastatic lymph nodes (LN) in rectal cancer.

**Methods:**

MEDLINE library, EMBASE, and SCOPUS database were screened for associations between DWI and metastatic and non-metastatic LN in rectal cancer up to February 2021. Overall, 9 studies were included into the analysis. Number, mean value, and standard deviation of DWI parameters including apparent diffusion coefficient (ADC) values of metastatic and non-metastatic LN were extracted from the literature. The methodological quality of the studies was investigated according to the QUADAS-2 assessment. The meta-analysis was undertaken by using RevMan 5.3 software. DerSimonian, and Laird random-effects models with inverse-variance weights were used to account the heterogeneity between the studies. Mean DWI values including 95% confidence intervals were calculated for metastatic and non-metastatic LN.

**Results:**

ADC values were reported for 1376 LN, 623 (45.3%) metastatic LN, and 754 (54.7%) non-metastatic LN. The calculated mean ADC value (× 10^−3^ mm^2^/s) of metastatic LN was 1.05, 95%CI (0.94, 1.15). The calculated mean ADC value of the non-metastatic LN was 1.17, 95%CI (1.01, 1.33). The calculated sensitivity and specificity were 0.81, 95%CI (0.74, 0.89) and 0.67, 95%CI (0.54, 0.79).

**Conclusion:**

No reliable ADC threshold can be recommended for distinguishing of metastatic and non-metastatic LN in rectal cancer.

## Introduction

Rectal cancer (RC) is the second most commonly diagnosed cancer among both men and women in the USA with more than 40,000 cases per year [1]. The presence of nodal metastases is one of the most important prognostic factors in rectal cancer. So far, it has been shown that patients with pN2 nodal involvement have worse survival [2, 3]. Therefore, the presence of lymph node metastases is acknowledged to predict overall survival (OS) and disease-free survival (DFS) in non-metastatic RC [2, 3]. Moreover, lymph nodal status is an essential factor in determining the need for adjuvant chemotherapy after surgical resection [2, 3]. Therefore, early and correct diagnosis of lymph node metastasis should improve assessment of the tumor stage and facilitate selection of the most appropriate treatment.

For staging purposes, magnetic resonance imaging (MRI) plays an essential diagnostic role in RC [4]. MRI has a high accuracy for tumoral (T) staging in RC [4]. However, for LN staging, the role of MRI is limited due to several concerns [5, 6]. It is related to the fact that classical morphological features like shape, size, signal intensity, and enhancement of contrast medium cannot reliably discriminate metastatic and non-metastatic LN. In fact, previous studies showed that the diameter of benign and malignant nodes in RC was similar, which leads to a low accuracy [5, 6]. Other morphological criteria, such as signal intensity, board margin, and enhancement intensity, did not improve significantly the diagnostic accuracy of metastatic LN in RC [6].

Some reports showed that diffusion weighted imaging (DWI) has a great diagnostic potential and can better characterize tumors than conventional MRI [7]. DWI is a magnetic resonance imaging (MRI) sequence based on the quantification of water motion in tissues which can be expressed by apparent diffusion coefficient (ADC) [7]. It has been widely shown that ADC is inversely associated with cell count and proliferation potential throughout oncology [7–9]. A key fact is that typically malignant tumors have lower ADC values in comparison to benign ones, which was shown for several body regions [10–12].

Presumably, due to the association with cellularity and microvasculature, DWI may be helpful to distinguish metastatic from non-metastatic LN as it could reflect distinctive histopathology differences between these.

However, there is still lack of reliable data regarding the accuracy of DWI to predict nodal status in RC.

Therefore, the purpose of the present meta-analysis was to evaluate diagnostic utility of DWI/ADC parameters for distinguishing metastatic and non-metastatic lymph nodes in RC.

## Methods

### Data acquisition

MEDLINE library and SCOPUS database were screened for associations between ADC and LN status in patients with RC up to February 2021. The following search terms/combinations were used as follows:

“DWI or diffusion weighted imaging or diffusion-weighted imaging or ADC or apparent diffusion coefficient AND rectal cancer OR rectal carcinoma OR rectum cancer OR rectum carcinoma AND lymph node OR lymph node metastases OR lymph node metastasis.” Secondary references were also manually checked and recruited. The Preferred Reporting Items for Systematic Reviews and Meta-Analyses statement (PRISMA) was used for the research [13].

**Fig. 1:**
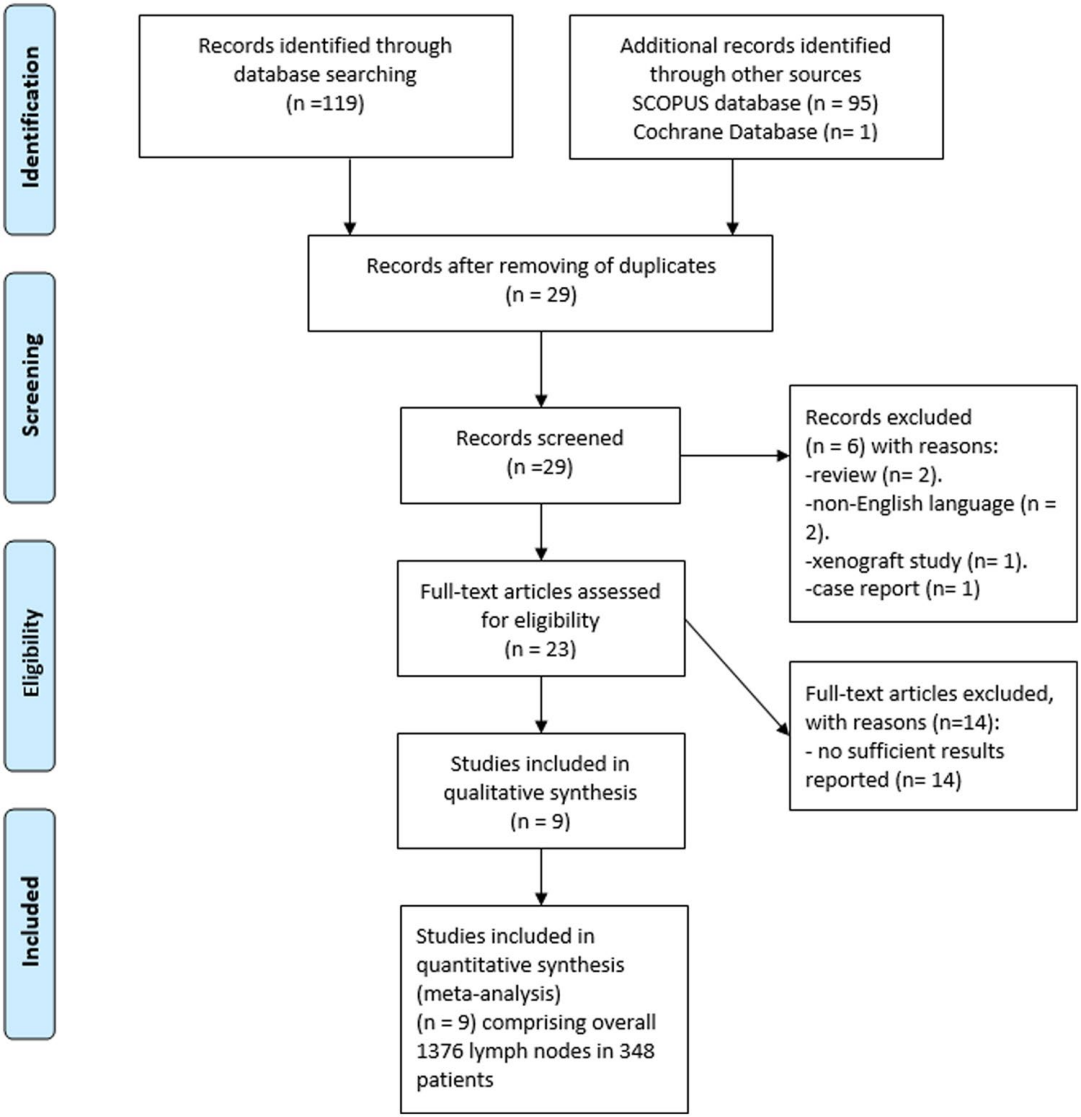
PRISMA flow chart of the data acquisition. Overall, 9 studies comprising with 1376 lymph nodes were included into the present study

The primary search identified 215 records (Fig. 1). The abstracts of the items were checked. Inclusion criteria for this meta-analysis were as follows:

- data derived from diffusion weighted imaging (DWI);
- available mean and standard deviation values of ADC;
- original studies investigated humans;

Exclusion criteria were as follows:

- studies unrelated to the research subjects;
- studies with incomplete data;
- duplicate publications;
- experimental animals and in vitro studies;
- review, meta-analysis and case report articles;

After thoroughly review, 9 items met the inclusion criteria and were included into the present analysis [14–22].

The following data were extracted from the literature: authors, year of publication, study design, number of patients, number of analyzed lymph nodes, mean value, and standard deviation of DWI parameters.

## Meta-analysis

**Fig. 2:**
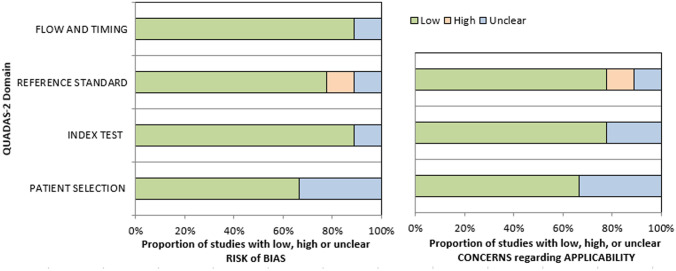
QUADAS-2 quality assessment of the included studies. Most studies showed overall a low risk for bias

On the first step, the methodological quality of the included 9 studies was checked according to the Quality Assessment of Diagnostic Studies (QUADAS-2) instrument [23] by one observer (H.J.M) (Fig. 2). On the second step, the reported DWI values (mean and standard deviation) were acquired. On the third step, the meta-analysis was undertaken by using RevMan 5.3 (RevMan 2014. The Cochrane Collaboration Review Manager Version 5.3.) [24, 25]. Heterogeneity was calculated by means of the inconsistency index I^2^. The interpretation of I^2^ was the following: 0 to 40%: not important, 30 to 60%: moderate heterogeneity, 50 to 90%: substantial heterogeneity, and 75 to 100%: considerable heterogeneity as defined by the Cochrane institute [26]. Then, DerSimonian and Laird random-effects models with inverse-variance weights were used without any further correction to account for the heterogeneity between the studies [27]. Mean values including 95% confidence intervals were calculated separately for metastatic and non-metastatic LN.

## Results

Of the included 9 studies, 2 were retrospective (22.2%), 5 prospective (55.6%), and in 2 studies (22.2%); the design was unclear (Table 1). Data regarding technical details of MR investigations are given in Table 2.

**Table 1:** Data regarding included studies

Authors	Study design	Patients	Investigated LN, total	Metastatic LN, n (%)	Reference standard
Cerny et al. [14]	Prospective	27	63	44 (69.8)	PET
Cho et al. [15]	Retrospective	34	114	46 (40.4)	Histopathology
Ge et al. [17]	Prospective	46	67	43 (64.2)	Histopathology
Heijnen et al. [16]	Retrospective	21	102	12 (11.8)	Histopathology
Li et al. [18]	Unclear	21	284	168 (59.2)	Histopathology
Qui et al. [19]	Prospective	68	160	93 (58.1)	Histopathology
Yasui et al. [20]	Prospective	46	162	76 (46.1)	Histopathology
Yu et al. [21]	Prospective	50	59	31 (52.5)	Histopathology
Zhuang et al. [22]	Unclear	35	115	65 (56.5)	Histopathology

*LN* lymph nodes

**Table 2:** Data about the involved patients and treatments

Patients	n (%)
Total	348
Female	166 (43.3)
Male	217 (46.7)
Age	
25–88 years	
Treatment	n (%)
Surgery	318 (83.0)
Neoadjuvant therapy and surgery	44 (11.5)
Not reported	21 (5.5)

### Risk of bias

Patient selection was generally well defined within the respective methodology; yet, 4 studies (44.4%) did not report the inclusion and exclusion criteria clearly which can account for potential bias.

All studies clearly reported methodology of the index test and were accordingly not considered a significant source of potential bias.

Eight studies (88.9%) utilized histopathology evaluations as reference test. Only one study can be considered as a risk of bias which used PET-CT as reference standard [14].

The acquired 9 studies comprised 348 patients with RC. Demographic data of the patients are shown in Table 3. In these patients, 1376 LN were analyzed. There were 623 (45.3%) metastatic LN and 754 (54.7%) non-metastatic LN.

**Table 3:** Technical details of MR investigations

Authors	MR scanner	b values, s/mm^2^	TR/TE, ms	FoV, mm	Slice thickness, mm
Cerny et al. [14]	1.5 T Magnetom Aera; Siemens Healthcare	0, 600	3200/55	350 × 563	5
Cho et al. [15]	1.5 T Signa Excite; GE Medical Systems	0, 1000	8000/85.2	300 × 300	5
Ge et al. [17]	3 T 750 W GE Medical Systems	0, 800	3648/70	360 × 360	5
Heijnen et al. [16]	1.5 T Intera or Intera Achieva; Philips Medical Systems	0, 500, 1000	4829/70	n.r	n.r
Li et al. [18]	Different 3 T scanners of GE Medical Systems	0, 50, 100, 150, 200, 300, 500, 800, 1000, 1300, 1500, 1700, 2000	2600/minimum	320 × 320	5
Qui et al. [19]	3 T Discovery 750, GE Medical Systems	0, 25, 50, 75, 100, 150, 200, 400, 600, 800, 1000, 1200, 1500, 2000	2200/minimum	260 × 260	4
Yasui et al. [20]	1.5 T Intera Philips Medical Systems	0, 800	3704/68	375 × 375	8
Yu et al. [21]	1.5 T Optima MR360, GE Healthcare	0, 10, 20, 30, 50, 80, 100, 150, 200, 400, 600, 800	4500/97	380 × 300	3
Zhuang et al. [22]	3 T	Unclear	Unclear	Unclear	Unclear

*n.r.* not reported

### ADC values of LN

**Fig. 3:**
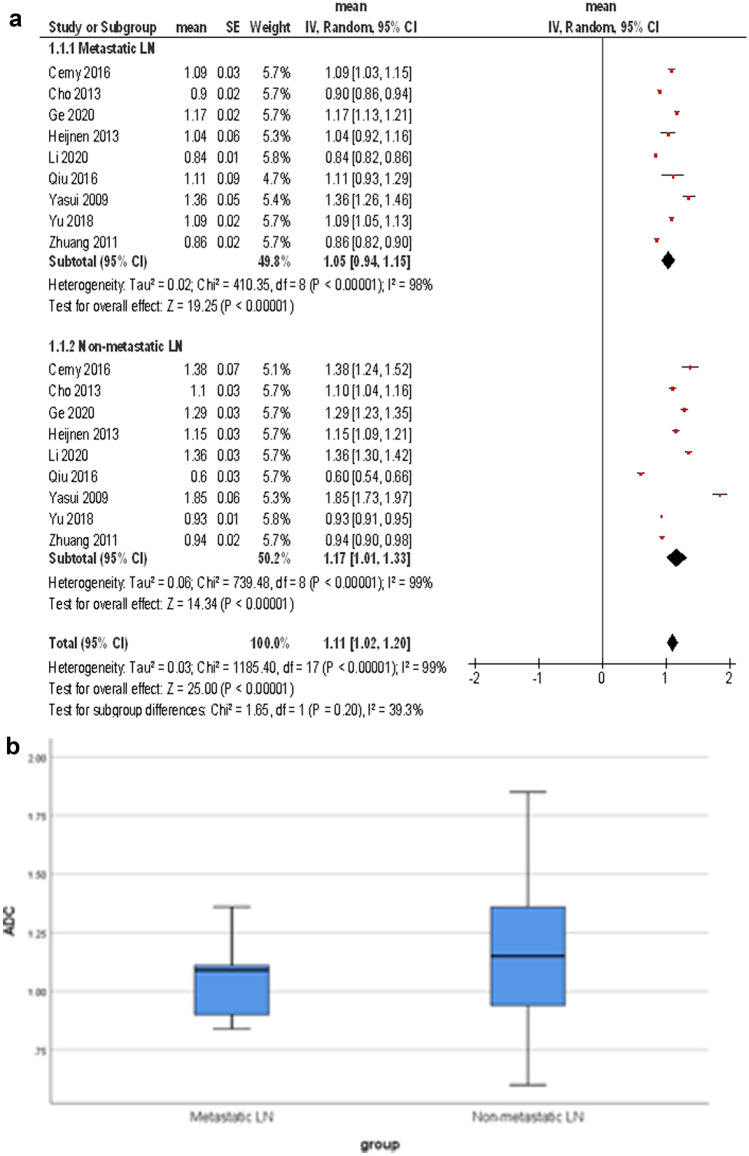
**a** Forest plots of ADC values reported for metastatic and non-metastatic lymph nodes. The calculated mean ADC value (× 10^−3^ mm^2^/s) of metastatic LN was 1.05, 95%CI (0.94, 1.15). The calculated mean ADC value of the non-metastatic LN was 1.17, 95%CI (1.01, 1.33). **b** Graphical distribution of ADC values of metastatic and non-metastatic lymph nodes. The box plots overlap significantly, that no clear threshold ADC-value can be recommended

ADC values were reported for 1376 LN, 623 (45.3%) metastatic LN, and 754 (54.7%) non-metastatic LN. The calculated mean ADC value (× 10^−3^ mm^2^/s) of metastatic LN was 1.05, 95%CI (0.94, 1.15). The calculated mean ADC value of the non-metastatic LN was 1.17, 95%CI (1.01, 1.33) (Fig. 3a). The graphical distribution of ADC values of metastatic and non-metastatic LN is shown in Fig. 3b.

### Tesla strength

A subgroup analysis was performed to divide the studies according to tesla strength.

**Fig. 4:**
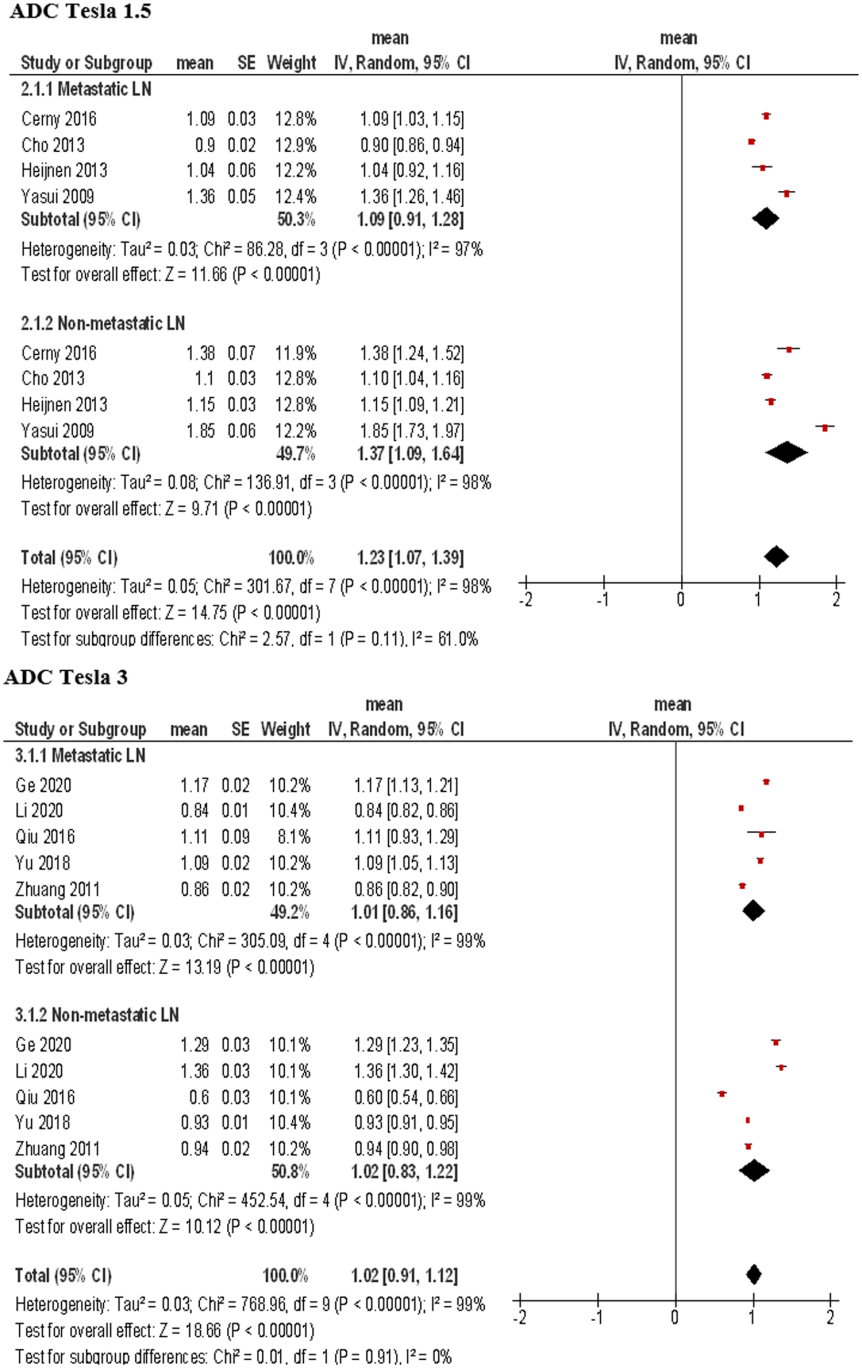
Forest plots of ADC values reported for metastatic and non-metastatic lymph nodes according to tesla strength. For 1.5 T scanners, the calculated mean ADC value (× 10^−3^ mm^2^/s) of metastatic LN was 1.09, 95%CI (0.91, 1.28), and of the non-metastatic LN, it was 1.37, 95%CI (1.09, 1.64). For 3 T scanners, the ADC value of metastatic LN was 1.01, 95%CI (0.86, 1.16), and for non-metastatic LN, it was 1.02, 95%CI (0.83, 1.22)

Four studies utilized a 1.5 T scanner comprising 441 LN, 178 metastatic LN (40.3%), and 264 non-metastatic LN (59.7%). The calculated mean ADC value (× 10^−3^ mm^2^/s) of metastatic LN was 1.09, 95%CI (0.91, 1.28). The calculated mean ADC value of the non-metastatic LN was 1.37, 95%CI (1.09, 1.64) (Fig. 4).

Five studies utilized a 3 T scanner comprising 935 LN, 445 metastatic LN (47.6%), and 490 non-metastatic LN (52.4%). The calculated mean ADC value (× 10^−3^ mm^2^/s) of metastatic LN was 1.01, 95%CI (0.86, 1.16). The calculated mean ADC value of the non-metastatic LN was 1.02, 95%CI (0.83, 1.22) (Fig. 4).

### Discrimination analysis

**Fig. 5:**
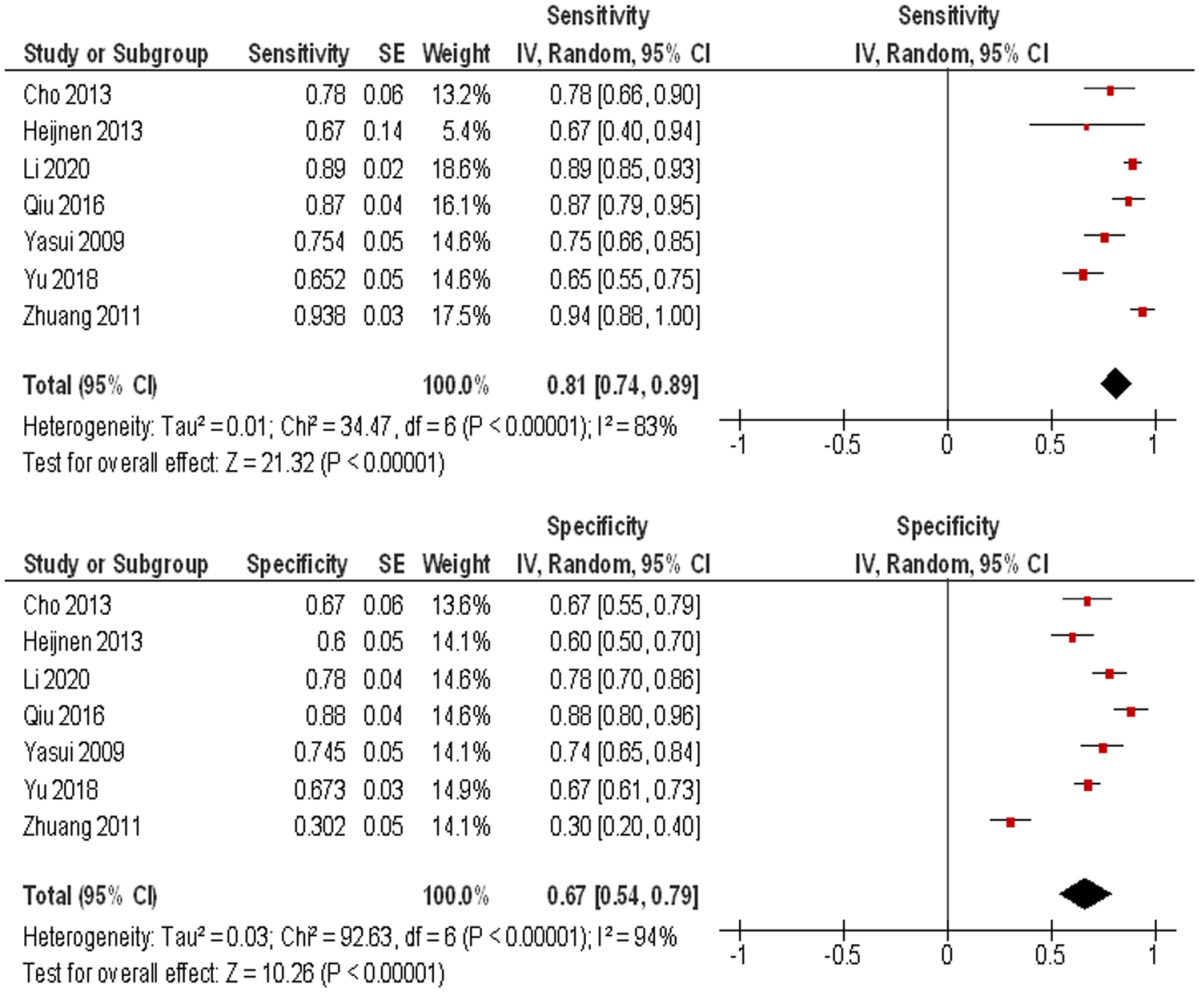
Forest plots of sensitivity (**a**) and specificity (**b**) of ADC values for distinguishing between metastatic and non-metastatic lymph nodes. The calculated sensitivity and specificity were 0.81, 95%CI (0.74, 0.89) and 0.67, 95%CI (0.54, 0.79), respectively

Furthermore, in 7 studies, ADC thresholds discriminating metastatic from non-metastatic LN and data of ROC analysis were reported (Table 4, Fig. 5).

**Table 4:** Optimal cutoffs, sensitivity, and specificity for ADC values

Autors	Cutoff ADC-values	Sensitivity	Specificity
Cho et al. [15]	1.00	0.78	0.67
Heijnen et al. [16]	1.07	0.67	0.60
Li et al. [18]	1.01	0.89	0.78
Qui et al. [19]	0.80	0.87	0.88
Yasui et al. [20]	1.44	0.75	0.74
Yu et al. [21]	0.98	0.65	0.67
Zhuang et al. [22]	1.05	0.93	0.30

The calculated sensitivity and specificity were 0.81, 95%CI (0.74, 0.89) and 0.67, 95%CI (0.54, 0.79), respectively (Fig. 5).

## Discussion

The present analysis addressed the important clinical question, whether DWI can aid to diagnose the correct nodal status in RC. This is of interest as on the one hand LN status plays a great prognostic role. On the other hand, conventional MRI cannot definitively discriminate metastatic and non-metastatic LN.

It is well known that lymph node metastases in RC occur usually along the mesorectal nodal chain of the inferior mesenteric artery, or in the lateral pelvic sidewall nodes, which include the internal iliac, obturator and medial external iliac chains [28, 29]. Interestingly, lateral pelvic lymph node metastases occur in 10–25% of patients with RC and are associated with higher local recurrence and reduced survival rates [28]. On the other hand, there is a significant risk of urinary and sexual dysfunction after surgical dissection of lateral pelvic lymph nodes [28, 29]. Therefore, recent studies indicated that a greater accuracy in preoperative staging is needed to select those patients that will benefit from lateral lymph node dissection surgery [29, 30].

These facts underline the need for imaging modalities and/or parameters, which can better identify LN metastases in RC.

As reported previously, besides diagnostic value, DWI can provide additional information regarding tissue microstructure [7–10]. One key finding is that ADC correlated inversely with cell count in different tumors [8]. Furthermore, ADC correlated also inversely with proliferation index Ki-67 indicating to reflect tumor biology [9]. So far, in ovarian cancer, cerebral lymphomas, and urothelial carcinoma, the pooled correlation coefficients between mean ADC and expression of Ki-67 were −0.62, −0.56, and −0.55, respectively [9]. In meningioma, ADC can differentiate low and high-grade tumors [31]. In prostate cancer, ADC is inversely associated with Gleason score and can be helpful to predict high-risk tumors [32].

DWI also reflects different histopathological features in RC. In the study of Ao et al., ADC correlated well with Ki-67 (r = −0.71, p < 0.01) [33]. Meng et al. showed that ADC correlated with expression of VEGF and HIF 1a [34].

Importantly, ADC can also distinguish malignant and benign lesions in different organs. For instance, in the head and neck region, it has been shown that ADC values ≤ 0.65 × 10^−3^ mm^2^/s had a positive predictive value of malignancy of 100% and ADC values ≤ 1.01 × 10^−3^ mm^2^/s had a positive predictive value of malignancy of 90% [35]. Furthermore, different breast cancers have typically ADC values lower than 1.00, whereas benign breast lesions have ADC values higher than 1.0 × 10^−3^ mm^2^/s [36]. Similar results were reported for renal lesions [37].

Previously, DWI parameters were also utilized for discrimination of benign LN from LN metastases throughout oncology. For example, Xing et al. showed that ADC value of metastatic LN was lower than non-metastatic LN in breast cancer with the high pooled sensitivity (0.86), specificity (0.86), PPV (0.82), and NPV (0.90) [38]. Similar results were also reported for ADC values in cervical LN [39].

In RC, the reported data were inconsistent. While some authors found that ADC could discriminate metastatic and non-metastatic LN in RC, others did not. For example, Cerny et al. found that mean ADC values of pathological LN were significantly lower than in control LN (p = 0.0012) [14]. Similar results were also reported by Heijnen et al. [16]. However, in the investigation of Qiu et al., metastatic LN showed higher ADC values (1.11 ± 0.89) in comparison to non-metastatic LN (0.6 ± 0.21), p < 0.01 [19].

Furthermore, the reported studies analyzed a relatively small number of patients and lymph nodes. These facts underline the need for evident data based on a large sample.

The present analysis shows that no reliable threshold for ADC values can be recommended to predict nodal status in RC. Another important point is the high heterogeneity identified of the ADC values. This might be caused by different scanner technology, b-values of the DWI, different ADC calculations, and field strength. This is crucial to acknowledged before ADC values can be used in clinical routine as a valuable imaging biomarker.

There are some limitations of the present study. First, it is based on published results in the literature with a known publication bias. Second, only a small number of studies met the inclusion criteria for this analysis and many studies were excluded because some data; e.g., ADC mean values and/or standard deviation were missing. Third, there is the restriction to published papers in English language. Fourth, different MR techniques, i.e., scanners, sequences, and slice thickness, were used in the included studies. Finally, in the included studies, some relevant clinical data like localization of the investigated lymph nodes, tumor stage, and grading were missing and could not be analyzed. Overall, the above mentioned factors resulted in a high heterogeneity between the studies. This fact may relativize our results. However, the results of this meta-analysis are based on a large cohort and provide evident data about the current role of DWI in LN staging in RC.

In conclusion, ADC cannot distinguish metastatic and non-metastatic LN in rectal cancer. No reliable ADC threshold can be recommended to predict nodal status.
